# Analytical evaluation of a fully automated immunoassay for faecal calprotectin in a paediatric setting

**DOI:** 10.11613/BM.2017.030710

**Published:** 2017-10-15

**Authors:** Britta Noebauer, Lejla Ramic, Andrea Konstantin, Christina Zachbauer, Elisa Einwallner

**Affiliations:** 1 Department of Laboratory Medicine, Medical University of Vienna, Vienna, Austria.; 2 Department of Pediatrics and Adolescent Medicine, Medical University of Vienna, Vienna, Austria.

**Keywords:** calprotectin, faeces, particle-enhanced turbidimetric immunoassay (PETIA), inflammatory bowel disease

## Abstract

**Introduction:**

Faecal calprotectin (FC) is a routinely used marker for identifying and monitoring children with inflammatory bowel disease (IBD). This non-invasive test is useful for screening children with gastrointestinal symptoms to avoid unnecessary invasive procedures. In this study, we validated for the first time the performance of a fully automated particle-enhanced turbidimetric immunoassay (PETIA) on the VITROS® 5600 analyzer for measurement of FC in symptomatic children and adolescents.

**Materials and methods:**

For performance validation of the PETIA (fCAL® turbo, Bühlmann Laboratories, Switzerland) on the VITROS® 5600 analyzer (Ortho Clinical Diagnostics, USA) limit of quantitation (LoQ), linearity, precision data and calibration curve stability were defined. Additionally, 95 faecal samples were measured using the PETIA, an enzyme-linked immunosorbent assay (ELISA; fCAL®, Bühlmann Laboratories, Switzerland) and a semi-quantitative lateral flow assay (Quantum Blue Reader®, Bühlmann Laboratories, Switzerland) for agreement evaluation.

**Results:**

The LoQ for calprotectin using PETIA on the VITROS® 5600 analyzer was 21 µg/g. The linearity range was 20 - 2100 µg/g and the precision study showed a total coefficient of variation (CV) between 2.3% and 8.9%. The calibration curve was stable for 4 weeks. Using the clinical samples quantifiable by PETIA, ELISA and the semi-quantitative lateral flow assay, Passing-Bablok regression analysis and Bland-Altman plots showed good agreement.

**Conclusions:**

Due to good performance characteristics and agreement with established methods, the fully automated PETIA on the routine chemistry analyzer VITROS® 5600 is a new analytical option for the rapid determination of FC.

## Introduction

Faecal calprotectin (FC) is a routinely used marker for identifying and monitoring children with inflammatory bowel disease (IBD). This non-invasive test is very useful for screening children with gastrointestinal symptoms and differentiating organic from functional intestinal disorders, to avoid unnecessary invasive procedures. The gold standard for diagnosis of IBD, endoscopic evaluation with ileocolonoscopy, can be very traumatizing, especially for children. Calprotectin, a 36 kDa protein, is predominantly located in the cytosol of neutrophil granulocytes (*1*). Besides, it is found in small parts in the cytosol of monocytes and macrophages, supporting the theory of having bactericidal and fungicidal properties (*2*). It is a marker of increased granulocyte turnover and consequently it is involved in the regulation of inflammatory reactions. Literature reported elevated calprotectin concentrations in plasma, synovial fluid, urine and faeces in inflammatory state (*3*). As it is upregulated in inflammatory gastrointestinal (GI) diseases, FC is used as diagnostic biomarker in the detection and observation of chronic inflammatory bowel diseases (IBD), for more than 10 years (*2*–*5*).

In paediatrics, FC is commonly used in the detection of newly active IBD, in monitoring of therapy success as well as in the prediction of relapse in IBD patients (*6*–*9*). With implementation of FC as biomarker in discriminating between functional and organic BD, the number of endoscopies has successfully been reduced in paediatrics (*10*). In addition, it is a very resistant protein and therefore a stable parameter for laboratory testing, as collected faeces can be sent to a laboratory and stored up to 7 days at room temperature prior to analysis (*11*, *12*). In healthy adult patients, FC concentrations < 50 µg/g faeces is considered to be normal, concentrations between 50 and 100 µg/g are treated as borderline while concentrations > 100 µg/g need more precise intervention and deeper clinical workup (*13*). Importantly, FC concentrations have been shown to vary with age, the highest concentrations reported in new-borns (*14*–*17*).

The initial procedure for measuring FC was to perform an enzyme-linked immunosorbent assay (ELISA). Disadvantage of this method is the long turnaround time, as a batch contains around 35-40 samples and, in reality, analysis is not started until a batch is full. For point-of-care (POC) diagnostics of single sample testing in short time (minutes), quantitative and semi-quantitative methods were established, but with the downside of a loss in accuracy compared to ELISA tests. For that reason, companies developed and launched new fully automated immunochemical methods with short test-turnaround times on existing routine chemistry analysers. Quite a number of laboratories use the VITROS^®^ 5600 analyzer, due to its small sample size needed, which is highly advantageous in paediatric settings. Therefore, we aimed to validate, for the first time, the recently launched Bühlmann fCAL^®^ turbo particle-enhanced turbidimetric immunoassay (PETIA) for FC measurement on the VITROS^®^ 5600 analyzer.

## Materials and methods

### Study design and subjects

Children and adolescents presenting to the ambulance of our hospital with gastrointestinal symptoms, were enrolled and retrospectively investigated for method validation at the paediatric laboratory of the Medical University of Vienna from November 2016 to April 2017. Subjects were included into investigation with the prerequisite of a doctor ordering a test for faecal calprotectin. Symptoms suggesting a test for intestinal disease included chronic diarrhoea, weight loss, abdominal pain, and cramping and/or mucous or bloody stool. A total of 95 stool samples of symptomatic children and adolescents were included, 56% males (N = 53) and 44% females (N = 42), with median age 10 years (range: 4 months – 18 years). The study was performed in accordance with the ethical principles of the Declaration of Helsinki and was approved by the local ethics committee (Ethics Committee of the Medical University of Vienna).

### Methods

Next to performance validation (which included limit of quantitation (LoQ), calibration curve stability, linearity and precision study) of the new PETIA (fCAL^®^ turbo, Bühlmann Laboratories, Switzerland) on the VITROS^®^ 5600 Integrated System (Ortho Clinical Diagnostics, New Jersey, USA), faecal calprotectin concentration measurements were compared to concentration values analysed by an ELISA test (fCAL^®^, Bühlmann Laboratories, Switzerland) and a semi-quantitative lateral flow assay (Quantum Blue Reader^®^, Bühlmann Laboratories, Switzerland). Therefore, FC concentrations in frozen faecal extracts were measured in duplicates by means of three different quantification methods. Diagnoses of ulcerative colitis (UC) and Crohn’s disease (CD) were confirmed by ileocolonoscopy and histology.

#### Sample extraction and preparation

Stool samples were extracted using the CALEX^®^ Cap device (Bühlmann Laboratories, Switzerland) according to the manufacturer’s instructions for sample preparation. Briefly, faecal samples were vortexed for 30 seconds and left for 10 minutes prior to analysis with the semi-quantitative lateral flow assay. After the initial determination with the semi-quantitative lateral flow assay for clinical purpose, faecal extracts were frozen at − 20°C. The anonymous frozen extracts, analysed for routine work, were then used for the validation of the PETIA on the VITROS^®^ 5600 Integrated system. Therefore, thawed faecal extracts were analysed in duplicates by the PETIA on VITROS^®^ 5600, with an ELISA test and the semi-quantitative lateral flow assay. For measurement with the PETIA and ELISA, the stool samples were additionally centrifuged for 10 min at 3000xg.

Regardless of the assay used for FC concentration measurement, all patient samples were measured in duplicates under same preanalytical conditions, using the arithmetic mean, for reflecting the imprecision for patient samples.

#### Quantum Blue Reader^®^

The point-of-care (POC) Quantum Blue Reader^®^ test is a semi-quantitative lateral flow immunoassay based on ELISA techniques. For performing the Quantum Blue Reader^®^ test, manufacturer’s instructions were followed. Briefly, a 60 µL aliquot of a single extraction sample was pipetted onto the test cartridge to allow lateral migration. After an incubation time of 12 minutes, absolute FC values were automatically generated and presented as µg/g in the dynamic range of 30 - 1000 µg/g.

#### Particle-enhanced turbidimetric immunoassay

The novel PETIA was performed on the VITROS^®^ 5600 Integrated System. The reagent kit (B-KCAL-RSET, fCAL® turbo, Bühlmann, Switzerland) contains a Reaction Buffer (R1) and Immunoparticles (R2). Human calprotectin from granulocyte extracts in Bühlmann extraction buffer was used to achieve six different calibrator levels (B-KCAL-CASET, fCAL® turbo Calibrator Kit, Bühlmann, Switzerland) as follows: 0, 52, 205, 509, 1023 and 2046 μg/g, respectively. The calprotectin concentration of the calibrator was assigned via a value transfer protocol toward Bühlmann fCAL^®^ ELISA. Controls (B-KCAL-CONSET, fCAL® turbo Control Kit, Bühlmann Switzerland) were prepared by adding human calprotectin from granulocyte extracts into Bühlmann extraction buffer, resulting in final concentrations of 75 μg/g (low) and 250 μg/g (high), respectively. A set of parameter settings was optimized for the immunoassay on VITROS^®^ 5600, being as follows:

Sample volume (μL): 10
R1 volume (μL): 130.0
R2 volume (μL): 26.0
Primary/secondary wavelength (nm): 575, and
Reading times (s): first reading 114 and second reading 175.75.

#### Enzyme-linked Immunosorbent Assay

For performing the Bühlmann fCAL^®^ ELISA, manufacturer’s instructions were followed as briefly summarized. After centrifugation of the prepared extract, for 10 minutes at 3000xg, the supernatant was diluted 1:50 with incubation buffer. A volume of 100 µL of the diluted supernatant was then transferred to an antibody-precoated ELISA plate and incubated for 30 minutes at room temperature. After a washing step, 100 µL of enzyme label was added prior to an additional 30 minutes incubation step. Additionally, 100 µL of substrate was pipetted to the 100 µL of enzyme label and incubated for another 15 minutes, protected from light. The optical density values were read at 450 nm on a spectrophotometer.

#### Validation of PETIA on the VITROS® 5600 Integrated System

##### Limit of quantitation

Limit of quantitation was defined as the lowest actual amount of the analyte that could be reliably detected with a total error below 20%. To assess the LoQ, a sample with an assigned value of 84 µg/g was diluted (1:4, 1:5 and 1:6, respectively) with extraction buffer, supplied from the manufacturer, for confirmation. All levels ranged from 14 to 21 μg/g. Using the known dilution factor and the assigned sample value, the theoretical concentration of the diluted samples could be calculated. The diluted samples were then aliquoted, frozen at − 20°C and measured in 12 replicates during 3 days, which resulted in 36 measurements for each concentration level in total. Biases from theoretical values were assessed and total error results were defined by the root mean square (RMS) method.

##### Linearity

For defining the linearity range, a high FC sample was diluted with distilled water in different concentrations, which resulted in eleven levels in a range from 100% - 0%. Concentrations above the highest calibrator were diluted 1:10 with distilled water, by the analyzer specific protocol and tested. All measurements were carried out in duplicates to calculate the deviation from the expected value. Recovery was calculated by comparing the observed values with the expected values.

##### Precision

For precision study, two controls and four faecal extracts (calprotectin concentration of the extracts were assigned via a value transfer protocol toward Bühlmann fCAL^®^ ELISA) were measured during 20 days, with two runs per day, and two replicates per run: a 20 x 2 x 2 design in accordance with CLSI protocol EP5-A3 (*18*). The coefficients of variation (CVs) for precision study were calculated within run (CVs in a single run without interruption; µg/g), between run (CVs between two runs per day after a minimum of 2 hours break; %), between days (CVs between days; %) and total (overall CV; %).

#### Calibration curve stability

First, calibration with the Calibrator Kit was carried out, which comprises blood-derived human calprotectin and is standardized against internal reference material. For baseline values (week 0), 4 stool extracts with validated calprotectin concentrations were measured 3 times in 2 runs, which resulted in 6 results per extract. Subsequently, the four stool extracts were measured in triplicates every consecutive week. The mean values of the measured calibrators per week were compared to the baseline values, to calculate the recoveries with an acceptance of 20% deviation from baseline. The study ended when recovery was out of range on two consecutive weeks.

#### Sample carry-over

There was no need for sample carry-over analysis as only disposable cuvettes and tips were used, for both the reagents and samples, on the clinical analyzer VITROS^®^ 5600. For that reason, the risk of carry-over on this analyzer is minimized.

### Statistical analysis

A total number of 95 stool samples for PETIA and ELISA and 53 stool samples for PETIA and the semi-quantitative assay were included for the agreement assessment between the different methods. Concentrations above the highest calibrator were diluted 1:10 with distilled water. Using the mean concentrations calculated from the duplicate measurements of each sample, Passing–Bablok regression analysis and Bland-Altman plots were performed from the PETIA with the mean concentration measured by the ELISA and the semi-quantitative lateral flow assay using MedCalc statistical software version 11.6 (MedCalc, Ostend, Belgium).

## Results

For the LoQ-analysis, samples in the concentration range of 14 to 21 µg/g were measured in 12 replicates during 3 days. Limit of quantitation was defined as the lowest concentration possible that showed a total error below 20%. The PETIA showed a LoQ of 21 μg/g calprotectin, on the VITROS^®^ 5600 analyzer, with a total error of 18.98%.

**Figure 1:**
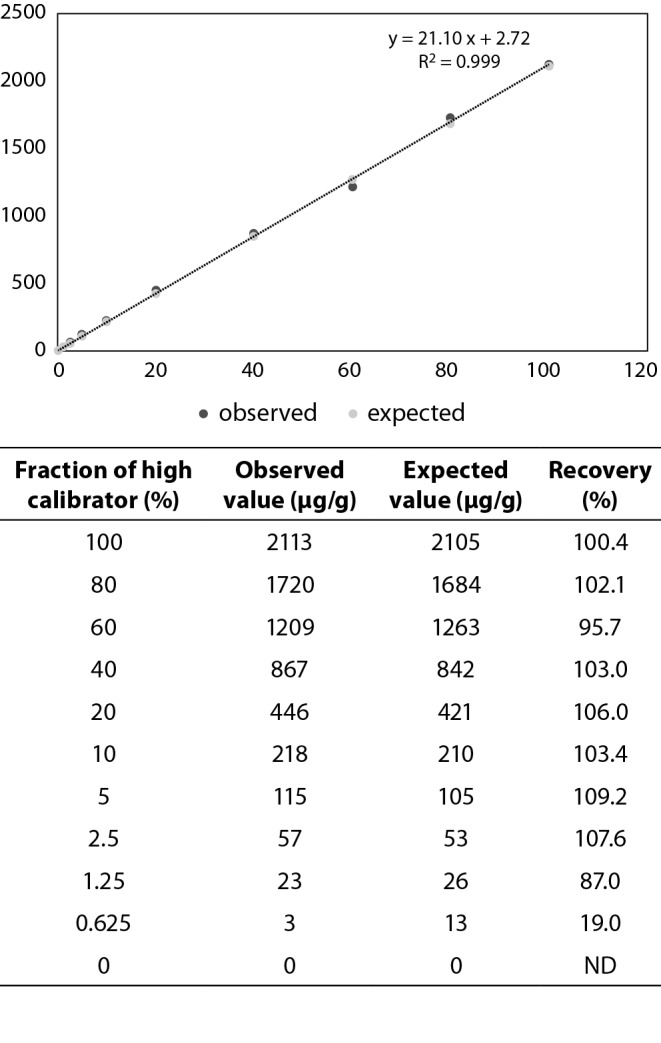
Linearity testing of the fully automated particle-enhanced turbidimetric immunoassay (PETIA) on the VITROS^®^ 5600. Linearity was tested and confirmed in the range from 20 to 2100 μg/g. ND – not detected.

For analysing the linearity of the PETIA, expected values were compared to the observed ones and recovery calculated. Results of linearity testing are listed in Figure 1 and confirmed linearity in the range from 20 - 2100 µg/g, the LoQ and the highest calibrator standard used.

For precision of the assay, within run CVs (2.15% - 6.63%), between day CV (< 0.01% - 4.72%), between run CV (< 0.01% - 6.05%) and total CV (2.33% - 8.89%) were calculated, details are shown in Table 1. Therefore, the expected imprecision was below 8.9% for sample concentrations between 50.6 and 1079 µg/g.

**Table 1:** Precision data analysis for the fully automated particle-enhanced turbidimetric immunoassay (PETIA) on VITROS^®^ 5600

	**Sample 1**	**Sample 2**	**Sample 3**	**Sample 4**	**Control low**	**Control high**
**Mean**	50.6	82.6	277.5	1078.7	77.0	243.8
**Within run CV (µg/g)**	6.63	2.47	0.97	0.46	5.10	2.15
**Between day CV (%)**	< 0.01	4.72	2.81	2.82	1.35	0.90
**Between run CV (%)**	6.05	3.10	2.63	1.17	< 0.01	< 0.01
**Total CV (%)**	8.89	6.16	3.97	3.09	5.27	2.33
Four distinct sample concentrations and two controls were used for the assessment of different coefficients of variations (CVs).						

Calibration curve stability testing revealed that the calibration curve was stable for a period of 4 weeks. The testing covered the range of expected results, using four different calprotectin concentration levels: 50, 80, 250 and 1000 µg/g. The samples measured in week 4 showed a recovery from baseline between 98% and 106%.

**Figure 2:**
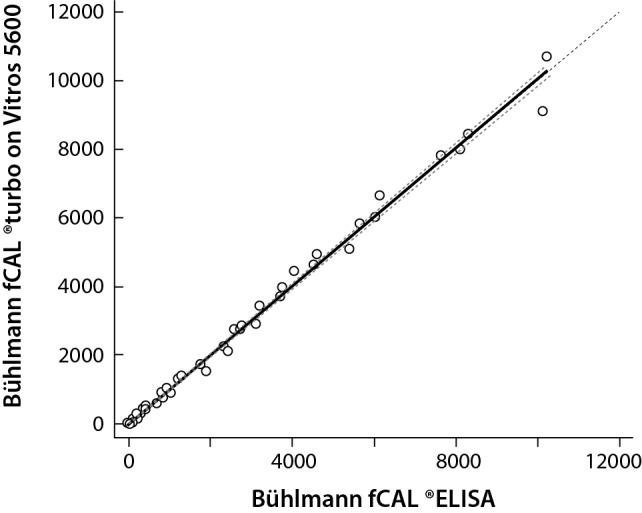
Passing-Bablok regression analysis of the fully automated particle-enhanced turbidimetric immunoassay (PETIA) performed on the VITROS® 5600 analyzer and an ELISA assay for faecal calprotectin. N = 95; concentration range: PETIA = 0 – 10,692 µg/g, ELISA = 0 – 10,237 µg/g; Pearson correlation coefficient R = 0.99; P < 0.001. Regression line equation with corresponding 95% CI for intercept and slope was y = 1.53 (- 0.69 to 3.14) + 1.01 (0.99 to 1.02) x. Cusum test for linearity indicates no significant deviation from linearity (P = 0.22).

**Figure 3:**
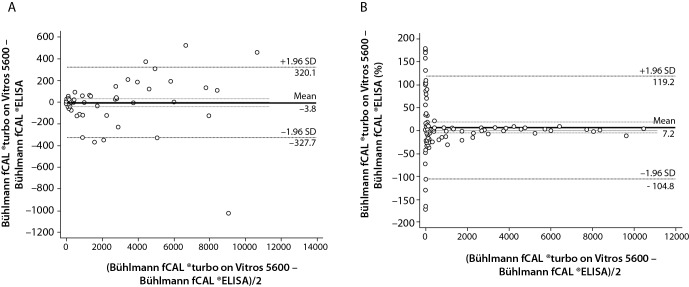
Bland-Altman plot for (A) absolute and (B) relative differences between the automated particle-enhanced turbidimetric immunoassay (PETIA) on the VITROS® 5600 analyzer and an ELISA assay for faecal calprotectin. The bold solid line represents the mean bias, the dashed lines the upper and lower limit of agreement. The grey lines represent the 95% confidence interval.

**Figure 4:**
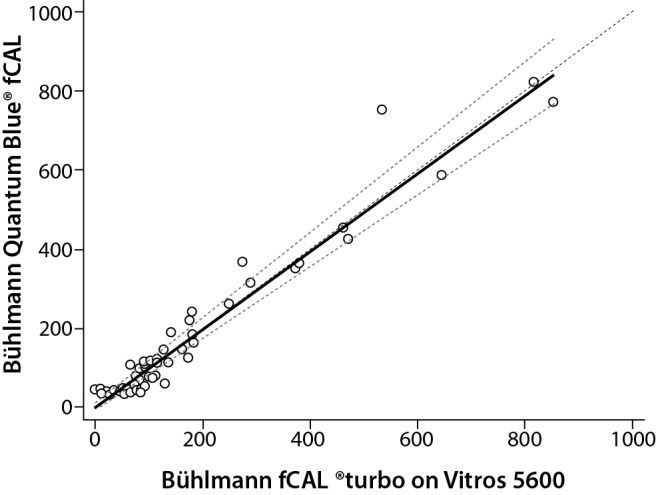
Method comparison of the automated particle-enhanced turbidimetric immunoassay (PETIA) performed on the VITROS® 5600 analyzer and a semi-quantitative lateral flow assay for faecal calprotectin. N = 53; concentration range: PETIA 0 – 852 µg/g, semi-quantitative lateral flow assay 31 – 820 µg/g. Pearson correlation coefficient R = 0.93, P < 0.001. Regression line equation with corresponding 95% CI for intercept and slope was y = 0.35 (- 10.03 to 7.38) + 0.99 (0.91 to 1.08) x. Cusum test for linearity indicates no significant deviation from linearity (P = 0.28).

**Figure 5:**
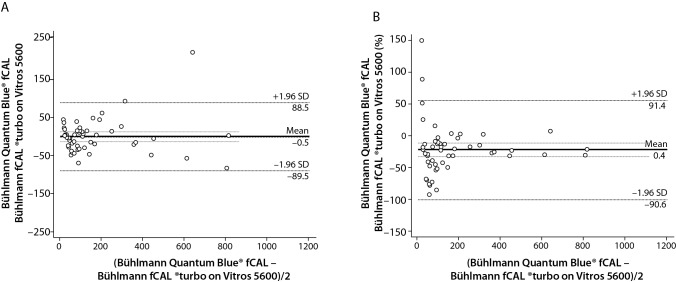
Bland-Altman plot: (A) absolute and (B) relative differences between the automated particle-enhanced turbidimetric immunoassay (PETIA) on the VITROS® 5600 analyzer and the semi-quantitative lateral flow assay for faecal calprotectin. The bold solid line represents the mean bias, the dashed lines the upper and lower limit of agreement. The grey lines represent the 95% confidence interval.

Analytical method agreement (Passing-Bablok regression analysis) and possible systematic bias (Bland-Altman plot) were investigated for the fully automated PETIA and the ELISA (Figure 2 and 3), and PETIA and the semi-quantitative lateral flow analyzer (Figure 4 and 5).

The Passing-Bablok linear regression analysis of the PETIA on VITROS^®^ 5600 and ELISA (N = 95) showed good agreement (R = 0.99), with an intercept (95% confidence interval, CI) of 1.53 (- 0.69 to 3.14) and a slope (95% CI) of 1.01 (0.99 to 1.02) (Figure 2). The FC concentrations of the faecal samples ranged from 0 to 10,692 µg/g by PETIA and from 0 to 10,237 µg/g by ELISA. In the Bland-Altman plots mean absolute and relative biases with corresponding limits of agreement were - 3.8 (- 327.7 to 320.1) (Figure 3A) and 7.2% (- 104.8 to 119.2) (Figure 3B), respectively.

The Passing-Bablok regression analysis for the PETIA and the semi-quantitative lateral flow assay (N = 53) illustrated acceptable agreement as well (R = 0.93), with an intercept (95% CI) of 0.35 (- 10.03 to 7.38) and slope (95% CI) of 0.99 (0.91 to 1.08) (Figure 4). The FC concentrations of faecal samples, used for method validation, ranged from 0 µg/g to 852 µg/g for the PETIA and from 31 µg/g to 820 µg/g for the semi-quantitative lateral flow assay. The Bland-Altman plot showed a mean absolute and relative bias with corresponding limits of agreement of - 0.5 (- 89.5 to 88.5) (Figure 5A) and 0.4% (- 90.6 to 91.4) (Figure 5B), respectively.

## Discussion

For rapid analysis of FC as a screening-parameter in IBD, an accurate assay with an easy, fast and good performance is appreciated. For that reason, we validated the performance of the fully automated PETIA on the VITROS^®^ 5600 integrated system, which is often used in paediatric laboratories because of its requires small sample volume (*19*).

Overall, the immunoassay performed well in terms of LoQ, linearity, precision and calibration curve stability. These results are in line with a recent study of Nilson *et al.* who evaluated the fully automated PETIA fCAL^®^ turbo for the analysers Mindray BS-380 and Cobas c501 (*20*). The study showed an equal linearity range for all three methods and similar LoQs for the PETIA on the VITROS^®^ 5600 and the Cobas c501 with 20 µg/g (BS-380: 11 µg/g). The precision was found with a total coefficient of variation between 3% and 8% in the 50 – 100 µg/g range for both analysers, respectively, and is in agreement with our results of the PETIA on the VITROS^®^ 5600. However, the BS-380 demonstrated stability for a period of 10 weeks, whereas the calibration curve for the fully automated PETIA on VITROS^®^ 5600 was stable for 4 weeks.

For method validation, we additionally compared the fully automated PETIA with the most commonly used ELISA assay (Bühlmann fCAL^®^ ELISA) and with a POC-method (Bühlmann Quantum Blue Reader^®^). The fully automated PETIA showed good agreement with the ELISA, as well as with the semi-quantitative assay. As already pointed out, the standard ELISA-based methods are generally used as reference methods for FC measurement, albeit they are labour intensive, time-consuming and have long test-turnaround times. As a result, many laboratories use the point-of-care desktop device with its lateral flow technology. Advantage is the possibility of single reliable results, obtained in less than 30 minutes, with simplicity of sample preparation and analysis. However, it is a semi-quantitative assay with a limited measurement range (*21*). In agreement with recent literature, the fully automated FC analysis methods turn out to be the future in routine laboratory work, as they unite short turnaround times, single sample investigation, easy as well as fast handling, a high measuring range and cost-effectiveness (*12*, *20*, *22*).

Two recent studies showed that using different extraction devices and procedures resulted in variability between distinct assays (*23*, *24*). Hence, we compared all three methods using only one extraction device, as all three assays are compatible with the same sample preparation for analysis. In this study, the Calex^®^ Cap device was used for protein extraction, as it can be used with any stool consistency. However, De Sloovere *et al*. demonstrated that the stool consistency does not affect FC results notably, when commercial extraction devices are used (*22*). Therefore, those devices are eminently suited in routine laboratory practice as their performance is acceptable and comparable, they are very easy to handle, they reduce hands-on preparation time and weighting of stool samples can be excluded.

This is the first study investigating a fully automated PETIA for FC measurement on the routine analyzer VITROS^®^ 5600. This procedure demonstrated an excellent overall performance and met our minimum demands in terms of quality standards. With its fine performance characteristics, its strong correlation with the reference method ELISA and its convenient handling, it is a new analytical option for the rapid determination of FC. Hence, it is the perfect tool for screening paediatric patients with gastrointestinal symptoms and monitoring children with IBD.
